# The Influence of Sex and Age on Survival in Patients with Hepatocellular Carcinoma

**DOI:** 10.3390/cancers16234023

**Published:** 2024-11-30

**Authors:** Iuliana Pompilia Radu, Bernhard Scheiner, Jonas Schropp, Maria Gabriela Delgado, Birgit Schwacha-Eipper, Chaonan Jin, Jean-Francois Dufour, Matthias Pinter

**Affiliations:** 1 Department of Visceral Surgery and Medicine, Inselspital, Bern University Hospital, University of Bern, 3012 Bern, Switzerland; jonas.schropp@protonmail.com (J.S.);; 2 Division of Gastroenterology and Hepatology, Department of Medicine III, Medical University of Vienna, 1090 Vienna, Austriamatthias.pinter@meduniwien.ac.at (M.P.); 3 Liver Cancer (HCC) Study Group Vienna, Medical University of Vienna, 1090 Vienna, Austria; 4 Hepatology, Department for BioMedical Research, University of Bern, 3012 Bern, Switzerland; 5 Centre Digestive Diseases, 1003 Lausanne, Switzerland

**Keywords:** hepatocellular carcinoma, sex, age, survival

## Abstract

**Background and Aim:** Age and biological sex are risk factors for hepatocellular carcinoma (HCC) occurrence, but their impact on overall survival (OS) is a matter of debate. This study aims to investigate how sex and age at diagnosis, along with other associated factors (i.e., comorbidities, etiologies, therapy) impact OS in the HCC population. **Method:** Data from two HCC cohorts—a prospective registry from the University Hospital of Bern, Switzerland, and a retrospective registry from General Hospital Vienna, Austria—were combined and analyzed. Clinical and laboratory data were reviewed, and OS was compared using Kaplan-Meier curves and the log-rank test. Cox regression models with penalized splines were applied to examine how age at diagnosis influenced OS. **Results:** Of 1547 HCC patients, 1284 (84.1%) were male. Females were older (67 vs. 63 years, *p* < 0.001) and had lower rates of liver cirrhosis (*p* < 0.001), cardiac comorbidities (*p* < 0.001), and diabetes (*p* < 0.001). No significant difference in median OS between men and women was observed (18 months vs. 16 months, *p* = 0.304). Mortality risk increased with age, particularly between 60 and 70 years. However, after adjusting for treatment, the age effect became non-significant (*p* = 0.171). **Conclusions:** After controlling the model for therapy, neither age nor sex independently influenced OS in HCC patients, underscoring the impact of therapy on survival.

## Simple Summary

The current study explores whether age and biological sex, along with other factors (i.e., comorbidities, underlying causes of disease, treatment options) impact overall survival in patients with hepatocellular carcinoma (HCC). Data of two European HCC cohorts were gathered and analyzed. Males represented the majority of the HCC population, were younger, and had more associated health issues than the female group. There was no significant difference in survival between males and females, but age seemed to influence survival, particularly for those aged 60–65. However, after considering treatment factors, the effect of age on survival was no longer significant, suggesting that therapy plays a crucial role in patient outcomes, regardless of age or sex.

## 1. Introduction

Hepatocellular carcinoma (HCC) is the most common primary liver malignancy and among the leading causes of cancer-related deaths globally [1]. Its incidence is rising, with an estimated 1.4 million new cases projected annually by 2040 [2]. Several new risk factors are responsible for the increasing burden of HCC (i.e., aging population, growing prevalence of diabetes, obesity, increased alcohol consumption) [3,4]. Despite the evolving landscape of therapy, the prognosis of these patients remains poor. The rising burden of HCC in the context of an aging population with more comorbidities and known discrepancies in therapy allocation within different groups underscores the need further research to better understand the influence of patient characteristics on therapy outcomes [5].

The male-to-female ratio of 4:1 confirms that male sex is a risk factor for HCC occurrence, but the impact of sex on survival remains a matter of debate [6]. This high incidence in the male population was attributed to hormonal and behavioral factors, such as higher testosterone levels, alcohol use, and higher rates of viral hepatitis in males [7]. Despite the higher incidence of HCC in males, some authors suggest that females may have a survival advantage, potentially due to the protective effects of estrogen [8,9]. The lack of advantage in survival in the female population over the male population, reported by some authors, was attributed to a different therapeutic approach in this population that is a result of several factors (i.e., older age at diagnosis, earlier stage at diagnosis, better Model for End-Stage Liver Disease (MELD) score) [10,11].

Age is another factor that negatively impacts survival in the elderly group. Patients over 65 years are categorized as elderly, and due to associated comorbidities, this group is less often assigned to radical therapies. Despite this, Guo H et al. showed that there was no difference between older and younger patients within similar Barcelona Clinic Liver Cancer (BCLC) stages receiving similar treatments [12].

In the present study, we aimed to investigate the relationship between sex, age at diagnosis, and overall survival in a large cohort of patients with HCC.

## 2. Materials and Methods

Data of a prospective HCC registry from University Hospital of Bern, Switzerland and a retrospective HCC registry from General Hospital & Medical University of Vienna, Austria were combined and analyzed. The Bern cohort comprised all HCC patients consecutively included in the prospective HCC cohort (*n* = 498 patients). The local ethics committee (Kantonale Ethikkommission Bern, Bern, Switzerland) approved collection of patient information and the study protocol (project-ID 2017-00957), which was consistent with the principles of the current version of the Declaration of Helsinki. Informed consent was obtained from all patients prior to study inclusion. The Vienna cohort included all patients with HCC consecutively included in the retrospective HCC registry (*n* = 1049). The local ethics committee of the Medical University of Vienna approved the collection of patient information and retrospective analysis of the data (EK # 1759/2015).

The following clinical data were retrieved from the databases of each cohort: patient demographics (age, sex), anthropometrics (height, weight), comorbidities (diabetes mellitus, arterial hypertension), etiology of underlying liver disease (chronic hepatitis B virus infection (HBV), chronic hepatitis C infection (HCV), alcohol-related liver disease (ARLD), metabolic-dysfunction-associated steatosis liver disease (MASLD), other etiologies (hemochromatosis, primary biliary cholangitis, primary sclerosing cholangitis, unknown etiology)), presence of cirrhosis (yes, no), Child-Pugh score, MELD score, BCLC classification, Eastern Cooperative Oncology Group (ECOG) score, treatment modality, and survival time.

In all cases, HCC diagnosis was established according to the diagnostic criteria of the European Association for the Study of the Liver (EASL) clinical practice guidelines [13]. The tumor stages were classified according to the BCLC staging system [14].

### 2.1. Statistical Analysis

Descriptive statistics are reported for the entire cohort and split by sex (male and female), age group (under 50 years, 50–60 years, 60–70 years, and over 70 years), and study center (Bern and Vienna). Baseline variables, including demographics, comorbidities, etiology, and disease and treatment information are reported as mean (SD), median [IQR], or *n* (%). Pearson’s chi-squared test and Fisher’s exact test are used to compare categories; Kruskal–Wallis and Wilcoxon rank sum tests are used to compare stochastic equivalence in continuous variables.

The cumulative incidence curves for overall survival (OS) were calculated to assess the potential influence of patient sex. Importantly, liver transplantation (LT) was included as a competing risk. To analyze the impact of age on OS, we employed various models. Each of these models uses penalized thin plate regression splines for flexible non-linear effects of age and other covariates and is adjusted for disease severity and patient sex at baseline. As patients were treated at several hospitals, we include a Gaussian random effect for the study center.

Initially, the Cox proportional hazards model for OS was applied utilizing age as a predictor. In order to test whether the effect of age on OS differs by sex, in the following model, we fit separate age effects for males and females and compared the two models using the likelihood ratio test.

In order to better understand how much (if any) of the effect of age on OS is due to treatment, we fit several models. For the first of these, patients were censored at the time of LT. This allowed us to discern the ‘direct effect’ of age on OS, disregarding the occurrence of LT. For the next model, LT was introduced as a time-dependent covariate. The data were segmented at each event and LT time, and a Cox-equivalent Poisson model was fitted because this method allows us to retain penalized splines for flexible non-linear effects of age (and other covariates). To enhance model flexibility, baseline hazards were allowed to vary based on transplant status. It is noteworthy that this model imposes additivity of predictor effects, which is more stringent compared to the model where patients are censored at LT. Additionally, we fit a model that includes type of treatment (LT/curative intention therapies (i.e., ablative, surgical resection)/palliative intention included systemic therapy (i.e., tirozin kinaze inhibitors (Sorafenib, Lenvatinib) and immunotherapy (Atezolizumab/Bevacizumab, Pembrolizumab, Nivolumab)) and locoregional therapies (chemoembolization (TACE), embolization (TAE), selective internal radiotherapy (SIRT), stereotactic body radiation therapy (SBRT)/best supportive care) integrated as a time-invariant variable. This allowed exploration of the impact of various treatments beyond LT but suffers from additional bias by discarding complex, time-dependent treatment information.

Results from a multinomial logistic regression analysis are presented, investigating the relationship between age and the type of treatment. This analysis aimed to provide further insight into the potential mediating role of treatment in the age-related effect on overall survival.

### 2.2. Supplementary Analyses

All models for the three primary research questions, (1) how OS in patients with HCC is related to sex, (2) how it is related to age, and (3) whether the association between age and OS differs between males and females, were fit again separately for each study center to compare the effects. The models and figures are largely identical but do not contain random effects.

## 3. Results

A total of 1547 patients with newly diagnosed HCC were analyzed, with the majority being male (*n* = 1284, 84.1%). The median age at diagnosis was 64 years (interquartile range [IQR]: 57–71), with a significant age difference between females and males (67 years vs. 63 years (*p* < 0.001)). Compared to males, females had a lower prevalence of liver cirrhosis (86% vs. 88%, *p* < 0.001), diabetes mellitus type 2 (23% vs. 36%, *p* < 0.001), arterial hypertension (33% vs. 40%, *p* = 0.025), and a lower BMI (25.6 vs. 26.6, *p* < 0.001). There was no significant difference in the etiology of liver disease between the sexes (*p* = 0.12). Both sexes had similar BCLC stages, while females had a slightly better MELD score (*p* = 0.019). Females less frequently received orthotopic liver transplantation (LT) (9.1% vs. 13%, *p* = 0.032) and were more frequently assigned to other curative therapies (i.e., ablative therapy, surgery) (33% vs. 28%, *p* = 0.032). Detailed information regarding the demographic and clinical characteristics of the analyzed cohort can be found in Table 1.

### 3.1. Association Between Age, Sex, and Median Overall Survival

**Figure 1:**
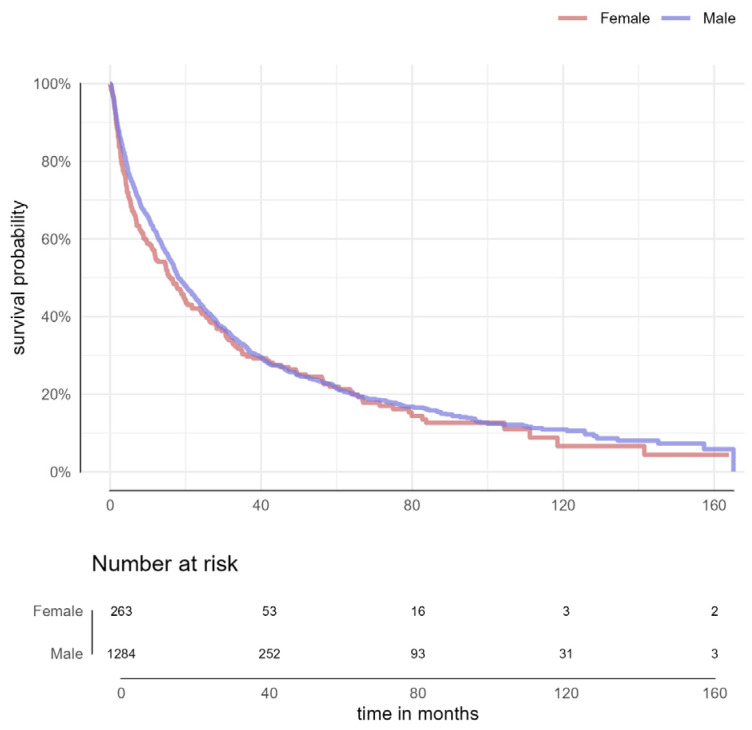
Overall survival of patients with hepatocellular carcinoma by sex.

For the entire cohort, the median OS was 17.89 months, 95% CI = [16.75; 19.89]. There was no statistically significant difference in OS between males and females (18 months for males [95% CI: 16.89–20.7] vs. 16 months for females [95% CI: 11.86–20.5], *p* = 0.304) (Figure 1).

The median survival times for the BCLC stages are as follows: BCLC 0–51.82 months [40.68–64.93], BCLC A 33.36 months [30.61–38.39], BCLC B 11.39 months [10.14–13.39], BCLC C 9.68 months [6.93–13.61], and BCLC D 3.25 months [2.57–4.61].

**Figure 2:**
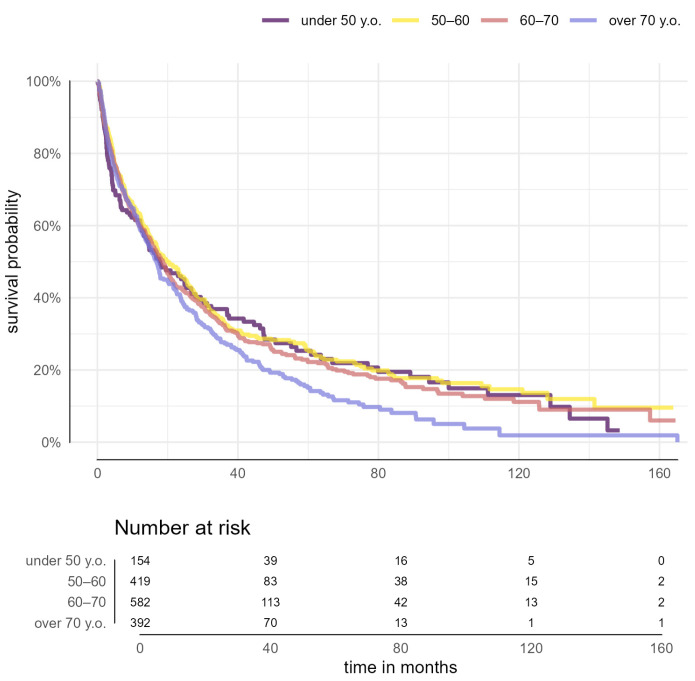
Overall survival by sex across age groups in patients with hepatocellular carcinoma (*p* = 0.104).

In the second step, the cohort was categorized into four groups (<50 years, 50–60 years; 60–70 years, >70 years). The risk of death increased progressively with each decade of life (*p* = 0.033) (Figure 2).

When comparing the two sexes within each age group by decade, no statistical difference in survival rates was noted (*p* = 0.104) (Table S1).

### 3.2. Effect of Age on Survival of the Patient

**Figure 3:**
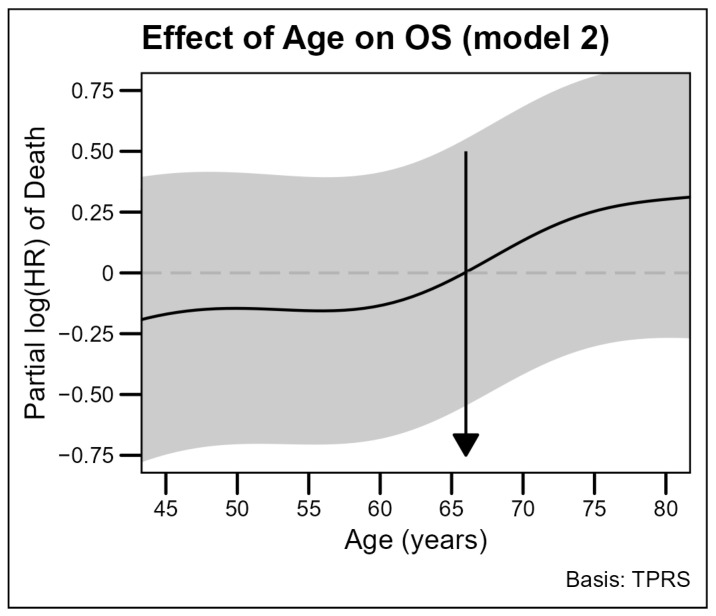
The effect of increasing age on overall survival in the adjusted model. The solid line represents the estimated log hazard ratios (log HRs) for age, pictures the relationship between patients’ age and overall survival (OS) in a cohort of 1514 patients. The impact of age on mortality risk was analyzed with a penalized spline (P-spline) model, treating patients’ age as a continuous covariate. Abbreviation: OS, overall survival, HR, hazard ratio.

Age was used as a continuous variable to assess the effect of age on survival. The mortality risk increased significantly with age without a clear cut-off, but more abruptly in the interval of 60–65 (edf = 3.91, *p* < 0.001) (Figure 3).

The coefficients and significance of the regression model are presented in Table S2. The model was tested in each sex and showed a stronger non-linear effect for males, while the effect for females appeared to be approximately linear. In both cases, the hazards of death were higher in older patients (Figure S1).

In the last step, we added the type of treatment in the model. The effect of age retained its directionality but became smaller and non-significant (*p* = 0.171), while the MELD score, therapy, and macrovascular invasion were independent factors associated with survival (Table 2).

### 3.3. Impact of Age on Treatment Selection

**Figure 4:**
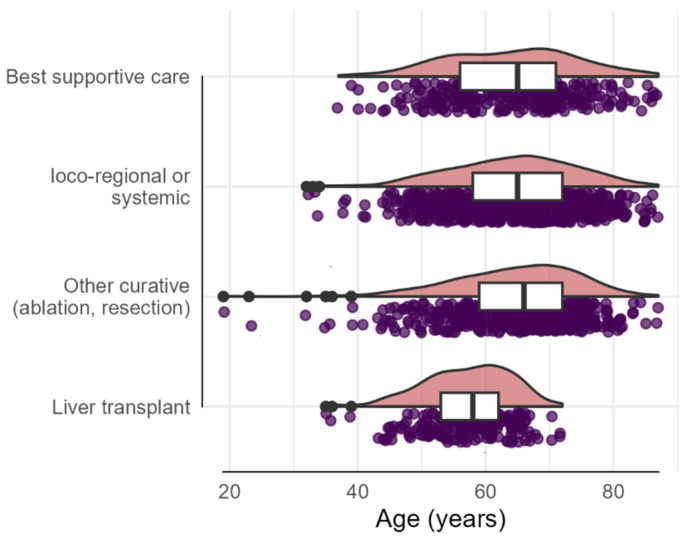
The distribution of treatment modalities across different age groups in HCC patients. On the *x*-axis is represented the patient age, and the *y*-axis lists the treatment: best supportive care, loco-regional (SIRT, TACE, TAE, SBRT) or systemic therapies (TKI, tyrosine kinase inhibitor (i.e., Sorafenib, Lenvatinib) and immunotherapy (i.e., nivolumab, pembrolizumab, atezolizumab/bevacizumab)), other curative therapies (ablation/resection), and liver transplantation. Each dot represents an individual patient. The pink density plots show the concentration of patients within each age range. The black line within the boxplots (white) indicates median age per group and the interquartile range (edges of the white boxes). The black dots represent outliers.

In our cohort, we observed that patients assigned to LT were younger than patients assigned to other curative treatments, and those receiving systemic treatments and best supportive care were older on average than those receiving an LT. Specifically, 21.5% of patients aged between 50 and 60 underwent OLT, while this percentage decreased to 11.5% for patients aged 60–70 (Figure 4).

## 4. Discussion

In this study, based on two large cohorts of HCC patients, despite differences in disease presentation between males and females, sex has no impact on survival. We found a non-linear relationship between age and mortality risk, with no clear age cut-off in the unadjusted model. This non-linear association persisted in the minimally adjusted model, which accounted for comorbidities, etiology, tumor stage, and liver function. After the model was further adjusted for therapy, the effect of age became statistically non-significant, indicating that treatment plays a critical role in survival of HCC patients.

Our results confirmed a higher incidence of HCC in the male population [6,10,15]. This preference for male sex was attributed to non-behavioral factors (i.e., genetic predispositions (e.g., overexpression of sex-determining region Y, low expression of long noncoding RNAs (LncRNA FTX)), sex hormones (lower estrogen levels, higher testosterone levels)), and behavioral factors (i.e., tobacco use, alcohol consumption, viral hepatitis infections) [16]. Although HCC is more prevalent in males than females across all regions, the 5:3 male-to-female ratio in our cohort likely reflects the higher-risk behaviors in the male population that contribute to the development of HCC [17].

The highest incidence of HCC was reported in Asian countries, where chronic HBV and HCV infections are the predominant etiologies [18]. Non-viral etiology predominated in our cohort, consistent with trends observed in Europe, where the viral etiologies were reported as being responsible for 10% of HCC cases. However, over 40% of patients in our cohort had HCC caused by HBV or HCV chronic infection, reflecting the ongoing relevance of viral causes in specific populations. In both Eastern and Western Europe, alcoholic liver cirrhosis is the most common background for HCC development, primarily affecting men and often occurring in patients without regular HCC surveillance [19,20]. Additionally, MASLD is rapidly becoming the fastest-growing cause of HCC, particularly in developed countries [17]. Regarding the relationship between sex and HCC risk in the MASLD population, several authors have noted a higher prevalence among males. However, Safcak D et al. reported an increased incidence in females. Factors such as postmenopausal age, obesity, and alcohol consumption were identified as contributing to this predisposition [21].

In the current cohort, females were diagnosed at an older age than males. The occurrence of HCC in the female population at an older age might be the result of the loss of protective effects of female sex hormones in the postmenopausal period [6,22]. In our cohort, no difference in tumor status at diagnosis was observed between the two groups, despite some authors reporting that females are diagnosed with HCC in earlier stages due to a better adherence to screening programs [6].

Despite males and females receiving curative HCC treatments at the same rate, females were less assigned to LT (9.1% vs. 13%, *p* = 0.032). These findings are consistent with other studies that showed that females have a longer waiting time and are less likely to receive LT compared with males [10,23]. Factors that might contribute to this disparity include older age at diagnosis, better MELD scores that may not accurately reflect liver function (i.e., lower muscular mass that results in a false lower creatinine), and shorter stature [10,11,24,25]. New MELD scoring systems have been proposed over time in order to reduce this incorrect assessment of liver function in women; however, there are required further adjustments that should address size mismatch for shorter candidates as well [26,27].

The lack of impact of sex on survival is consistent with previous studies, though it contrasts with others that have identified that females have better survival [10,11,28]. These discrepancies may be due to differences in study design, population characteristics (e.g., race, liver disease etiologies, liver dysfunction), or therapy.

Unlike prior studies that proposed specific age thresholds, we observed a non-linear association between age and mortality risk, without a distinct age cut-off [29,30]. Our findings align with those of a retrospective multicenter study from Central Europe, which reported no significant difference in overall survival between geriatric patients with HCC and younger controls, even in a subpopulation of patients without or with comorbidities [21]. The lack of effect of age in our fully adjusted model indicates that treatment choices have a major impact on survival. In this study, patients older than 60 years were less likely to undergo OLT than those under 60 years. The decision to assign older patients for LT remains complex, mainly due to organ scarcity and better long-term survival foreseen for young populations [31]. Dolnikov S suggested an age-based matching system could be a viable solution, with older grafts allocated to older recipients who may have shorter life expectancies [32]. However, this approach could lead to inequities, as younger recipients may face longer wait times due to the scarcity of younger donors [33]. Concerning systemic therapy in HCC patients, clinical trials indicate that available treatments are beneficial for elderly patients (≥75 years), with efficacy, toxicity, tolerability, and quality of life outcomes comparable to those of younger patients, provided their ECOG performance status is maintained at 0 or 1 [34]. In the light of our findings and literature data, we advocate an individualized approach based on factors such as cardiopulmonary comorbidities, nutritional status, frailty, and performance status, rather than using a strict age cut-off for treatment decisions. This approach is in line with the recommendations of the American Society of Clinical Oncology (ASCO), which advocates geriatric assessment in order to tailor the therapy [35].

Our study’s strengths include the large sample size and multicenter cohort, which provide robust data for analysis. Secondly, using age as a continuous variable in a flexible model, we captured nuanced interactions between age, treatment, and survival without losing critical information. Limitations include its retrospective design, which makes patient selection and confounding bias inevitable. In addition, the multicentric and retrospective design might be responsible for the nonuniformity of therapy criteria over the years and across the centers. Another limitation is the lack of detailed data on therapy-related side effects, such as bleeding or other complications, which may impact survival and long-term outcomes. However, the model was adjusted to account for various therapies, including liver transplantation, minimizing the impact of this limitation.

## 5. Conclusions

While patient sex and age do not appear to affect survival in HCC patients, differences in age at diagnosis, liver function, and treatment modalities between males and females suggest that individualized treatment strategies are needed. Future studies should explore whether alternative MELD scores (e.g., MELD-Na-Shift or MELD-GRAIL-Na) could address gender imbalances in liver transplantation. For patients over 60, a refined prognostic scoring system that incorporates comorbidities, frailty, liver disease severity, and cancer screening is needed to improve organ allocation and outcomes.

## Figures and Tables

**Table 1:** Demographic, clinical, and treatment characteristics of the pooled cohort.

Characteristic	Overall, N = 1547	Female, N = 263	Male, N = 1284	*p*-Value
**Age at diagnosis (median, IQR)**	64 (57–71)	67 (58–73)	63 (56–70)	**<0.001**
Age groups				**<0.001**
18–50	154 (10.0%)	25 (9.5%)	129 (10%)	
50–60	419 (27.1%)	54 (20.5%)	365 (28.5%)	
60–70	582 (37.6%)	88 (33.5%)	494 (38.5%)	
>70	392 (25.3%)	96 (36.5%)	296 (23.1%)	
**Etiology**				0.12
Virus (HBV or HCV)	632 (41%)	97 (37%)	535 (42%)	
ALD	432 (28%)	87 (33%)	345 (27%)	
MASLD	251 (16%)	36 (14%)	215 (17%)	
Unknown, PBC, HH	232 (15%)	43 (16%)	189 (15%)	
**MELD (median, IQR)**	9.2 (7.4–12.9)	8.8 (7.1–12.3)	9.3 (7.5–13.0)	**0.019**
**Comorbidities**				
Diabetes Mellitus (Yes)	521 (34%)	61 (23%)	460 (36%)	**<0.001**
Arterial Hypertension (Yes)	607 (39%)	87 (33%)	520 (40%)	**0.025**
HIV	7 (0.5%)	0 (0%)	7 (0.6%)	0.6
Unknown	53	9	44	
**Smoking**				**<0.001**
Never	306 (27%)	63 (38%)	243 (25%)	
Ongoing	426 (37%)	44 (26%)	382 (39%)	
Past	414 (36%)	61 (36%)	353 (36%)	
Unknown	401	95	306	
**BMI**	26.5 (23.9, 29.7)	25.6 (22.3, 29.2)	26.6 (24.1, 29.7)	**<0.001**
Unknown	100	20	80	
ECOG				0.2
0	571 (54%)	93 (51%)	478 (55%)	
1	129 (12%)	27 (15%)	102 (12%)	
2	277 (26%)	56 (30%)	221 (26%)	
3	66 (6.3%)	7 (3.8%)	59 (6.8%)	
4	6 (0.6%)	1 (0.5%)	5 (0.6%)	
Unknown	498	79	419	
**Cirrhosis (Yes)**	1354 (88%)	225 (86%)	1129 (88%)	0.3
**Child Pugh class**				0.6
A	636 (47%)	99 (44%)	537 (48%)	
B	474 (35%)	85 (38%)	389 (34%)	
C	244 (18%)	41 (18%)	203 (18%)	
**BCLC stage**				0.8
BCLC 0	223 (14%)	37 (14%)	186 (14%)	
BCLC A	453 (29%)	76 (29%)	377 (29%)	
BCLC B	490 (32%)	89 (32%)	417 (32%)	
BCLC C	109 (7%)	18 (19%)	91 (7.1%)	
BCLC D	256 (17%)	43 (16%)	213 (17%)	
**Therapy**				**0.032**
LT	190 (12%)	24 (9.1%)	166 (13%)	
Curative intent (resection, MWA, RFA)	447 (29%)	86 (33%)	361 (28%)	
Palliative intent (TAE, TACE, TARE, SBRT, systemic therapy (i.e., TKI, immunotherapy))	643 (42%)	97 (37%)	546 (43%)	
BSC	267 (17%)	56 (21%)	211 (16%)	

Abbreviations: IQR, interquartile range; BMI, body mass index; ARLD, alcohol-related liver disease; MASLD, metabolic-dysfunction-associated steatotic liver disease; HBV, hepatitis B virus; HCC, hepatocellular carcinoma; HCV, hepatitis C virus; PBC, primary biliary cholangitis; HH, hereditary hemochromatosis; AFP, alpha-fetoprotein; MELD, Model for End-Stage Liver Disease; BCLC, Barcelona Clinic Liver Cancer; OLT, orthotopic liver transplantation; TACE, transarterial chemoembolization; TARE, transarterial radioembolization; SBRT, stereotactic body radiation therapy; RFA, radiofrequency ablation; MWA, microwave ablation; TKI, tirozine kinase inhibitors; BSC, best supportive care. Footnotes: Bolded *p* values indicate significant difference (*p* < 0.05). Systemic therapy included TKI (i.e., Sorafenib, Lenvatinib) and immunotherapy (i.e., Nivolumab, Pembrolizumab, Atezolizumab/Bevacizumab).

**Table 2:** Multivariable analysis of risk factors associated with overall survival in patients with hepatocellular carcinoma. Model 3 adjusted for therapy.

Variable	HR	edf	*p*
Sex (male)	0.96	-	0.532
Presence of extrahepatic metastases (Yes)	1.10	-	0.239
Macrovascular involvement (Yes)	1.49	-	<0.001
BCLC stage A	1.28	-	0.026
BCLC stage B	2.00	-	<0.001
BCLC stage C	2.86	-	<0.001
BCLC stage D	2.32	-	<0.001
Curative intent (MWA, RFA, resection)	3.23	-	<0.001
Palleative intent (e.g., TACE, TAE, SIRT, systemic therapy (i.e., TKI, immunotherapy)	5.67	-	<0.001
Best supportive care	15.74	-	<0.001
Age (in years; tp)		1.01	0.193
MELD score (tp)		5.01	<0.001

Abbreviations: MELD, Model for End-Stage Liver Disease; BCLC, Barcelona Clinic Liver Cancer; LT, orthotopic liver transplantation; TACE, transarterial chemoembolization; TARE, transarterial radioembolization; SBRT, stereotactic body radiation therapy; RFA, radiofrequency ablation; MWA, microwave ablation; TKI, tyrosine kinase inhibitor; BSC, best supportive care; tp, penalized thin plate spline; HR, hazard ratio; edf, estimated degrees of freedom. Footnote: Systemic therapy included TKI (i.e., Sorafenib, Lenvatinib) and immunotherapy (i.e., Nivolumab, Pembrolizumab, Atezolizumab/Bevacizumab); Reference groups-Each HR compares the variable to its respective reference group (e.g., males vs. females; treatment vs. LT, each BCLC A, B, C, D vs. BCLC 0; Macrovascular involvement yes vs. no; presence of extrahepatic metastases yes vs. no). Age and MELD score were considered as continuous variables.

## Data Availability

Data are unavailable due to privacy.

## Supplementary Materials

The following supporting information can be downloaded at: https://www.mdpi.com/article/10.3390/cancers16234023/s1, Figure S1: Effect of Age on Overall Survival per each sex; Table S1: Sex-based comparison of overall survival across age groups; Table S2: Multivariable analysis of risk factors associated with overall survival in patients with hepatocellular carcinoma (Model 2).
