# Supplementary material for: The Influence of Sex and Age on Survival in Patients with Hepatocellular Carcinoma
Source: Cancers (Basel). 2024 Nov 30;16(23):4023. doi: 10.3390/cancers16234023 (PMC11640092; doi:10.3390/cancers16234023)
Supplement: Supplementary file 1 [file cancers-16-04023-s001.zip › Supplementary Figure S1.pdf]

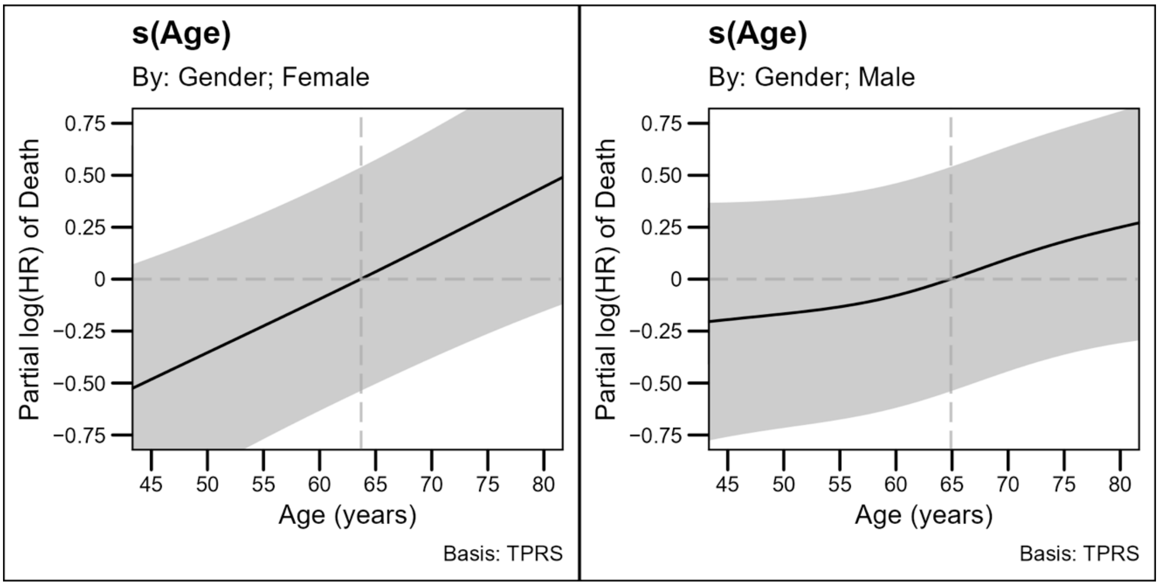

Supplementary Figure S1. Effect of Age on Overall Survival per each sex. The log hazard ratio (HR) of age on overall survival (OS) when fitting separate effects for men and women using an interaction effect. This figure illustrates how the effect of age on OS differs between sexes, emphasizing the interaction effect between age and sex in the survival analysis.
