# Supplementary material for: The Influence of Sex and Age on Survival in Patients with Hepatocellular Carcinoma
Source: Cancers (Basel). 2024 Nov 30;16(23):4023. doi: 10.3390/cancers16234023 (PMC11640092; doi:10.3390/cancers16234023)
Supplement: Supplementary file 1 [file cancers-16-04023-s001.zip › Supplementary Table S1.pdf]

Supplementary Table S1. Sex-based comparison of overall survival across age groups

Variables	under 50 years		50-60		60-70		>70	
Sex	Female	Male	Female	Male	Female	Male	Female	Male
n	25	129	54	365	88	494	96	296
Deaths	17	95	41	245	60	355	77	227
Median survival [95% CI]	15 [0–54.38	16.75 [12.61–27.93]	17 [10.66–23.35]	19 [14.79–23.20]	17 [7.64–26.31]	17 [14.72–19.22]	12.04 [5.28–16.72]	17.32 [12.08–17.91
p	0.640		0.550		0.758		0.150	

Abbreviation: number of patients analyzed, n; 95% confidence interval, 95% CI
