# Supplementary material for: The Influence of Sex and Age on Survival in Patients with Hepatocellular Carcinoma
Source: Cancers (Basel). 2024 Nov 30;16(23):4023. doi: 10.3390/cancers16234023 (PMC11640092; doi:10.3390/cancers16234023)
Supplement: Supplementary file 1 [file cancers-16-04023-s001.zip › Supplementary Table S2.pdf]

Supplementary Table S2. Multivariable analysis of risk factors associated with overall survival in patients with hepatocellular carcinoma (Model 2).

Variable	HR	edf	p
Sex(male)	0.92		0.3
Age (in years; tp)		4.06	.008
Presence of extrahepatic metastases (Yes)	1.49		<0.001
Macrovascular involvement (Yes)	1.98		<0.001
BCLC stage A	1.63		<0.001
BCLC stage B	2.44		<0.001
BCLC stage C	3.80		<0.001
BCLC stage D	3.25		<0.001
MELD score (tp)		5.74	<0.001
LT	0.43		<0.001
time since diagnosis (untransplanted; in months; tp)		1.31	0.222
time since diagnosis (transplanted; in months; tp)		2.12	0.006

Abbreviations: MELD, Model for End-Stage Liver Disease; BCLC, Barcelona Clinic Liver Cancer; LT, orthotopic liver transplantation; tp, penalized thin plate spline; HR, hazard ratio; edf, estimated degrees of freedom. Reference groups-Each HR compares the variable to its respective reference group (e.g., males vs. females; LT vs. non-LT, each BCLC A, B, C, D vs. BCLC 0; Macrovascular involvement yes vs. no; presence of extrahepatic metastases yes vs. no). Age and MELD score were considered as continuous variables. Time since diagnosis ('months') calculated as 28-day intervals for consistency.
